# Fibrin glue does not promote migration and proliferation of bone marrow derived mesenchymal stem cells in collagenic membranes: an in vitro study

**DOI:** 10.1038/s41598-022-25203-4

**Published:** 2022-11-30

**Authors:** Filippo Migliorini, Julia Prinz, Jörg Eschweiler, Hanno Schenker, Christian Weber, Nicola Maffulli, Sophie Lecouturier, Frank Hildebrand, Johannes Greven

**Affiliations:** 1 Department of Orthopaedic, Trauma, and Reconstructive Surgery, RWTH University Hospital, 52074 Aachen, Germany; 2 Department of Ophthalmology, RWTH University Hospital, 52074 Aachen, Germany; 3 Department of Medicine, Surgery and Dentistry, University of Salerno, 84081 Baronissi, SA Italy; 4 School of Pharmacy and Bioengineering, Keele University Faculty of Medicine, Stoke on Trent, ST4 7QB England; 5 Queen Mary University of London, Barts and the London School of Medicine and Dentistry, Centre for Sports and Exercise Medicine, Mile End Hospital, 275 Bancroft Road, London, E1 4DG England

**Keywords:** Biotechnology, Diseases

## Abstract

During Autologous Matrix-Induced Chondrogenesis (AMIC), the membrane is often glued into the chondral defect. However, whether fibrin glue influences cells proliferation and migration remain unclear. This study evaluated the impact of fibrin glue addition to biologic membranes loaded with bone marrow-derived mesenchymal stem cells (B-MSCs). A porcine derived collagen membrane (Cartimaix, Matricel GmbH, Germany) was used. B-MSCs were harvested from three different unrelated donors. The membranes were embedded in mounting medium with DAPI (ABCAM, Cambridge, UK) and analysed at 1-, 2-, 3-, 4-, 6-, and at 8-week follow-up. The DAPI ties the DNA of the cell nucleus, emitting blue fluorescence. DAPI/nuclei signals were analysed with fluorescence microscopy at 100-fold magnification. The group without fibrin glue demonstrated greater migration of the B-MSCs within the membrane at week 4 (P < 0.001), 6 (P < 0.001), and 8 (P < 0.001). No difference was found at week 1, 2, and 3. The group without fibrin glue demonstrated greater proliferation of B-MSCs within the membrane. These differences were significant at week 1 (P = 0.02), 2 (P = 0.008), 3 (P = 0.0009), 4 (P < 0.0001), 6 (P < 0.0001), 8 (P < 0.0001). Concluding, in the present setting, the use of fibrin in a collagenic biomembrane impairs B-MSCs proliferation and migration in vitro.

## Introduction

Chondral defects lead to chronic pain, reduced quality of life and sport activities, and osteoarthritis may result^1,2^. Conservative management often does produce long term improvement, and residual symptoms are common^3,4^. Bone marrow stimulating procedures have been advocated for chondral defects^5–7^, aiming to produce a continuum between subchondral bone and cartilage to enhance bone marrow-derived mesenchymal stem cells (B-MSCs) migration and proliferation into the chondral defect^3,8^. Autologous Matrix-Induced Chondrogenesis (AMIC) is a bone marrow stimulating procedure which demonstrated efficacy and safety for chondral defects of the knee and talus^9,10^. During AMIC, the blood clot from the microfractured subchondral bone is stabilised using a bioresorbable membrane. The fixation of this membrane into the defect can be problematic, and most clinical studies secured the AMIC membrane to the chondral defect using fibrin glue^11–14^. However, recently, the addition of fibrin glue to the membrane has been questioned. Several clinical studies on matrix-induced autologous chondrocyte implantation (mACI) demonstrated that the membrane remains stable even without formal fixation^15–19^. MACI is not considered a bone marrow stimulating procedure, as it delivers a membrane loaded with expanded autologous chondrocytes into the defect^9,20^. However, the nature of the membrane used in mACI is the same as in AMIC^21,22^. To the best of our knowledge, the impact of fibrin glue addition on B-MSCs migration and proliferation has not yet been investigated in vitro. The present investigation assessed the in vitro influence of the addition of fibrin glue to a B-MSCs loaded porcine derived collagen I/III membrane commonly employed in AMIC. The outcomes of interest were to compare migration and proliferation of B-MSCs with or without fibrin glue addition.

## Methods

### Study protocol

**Figure 1:**
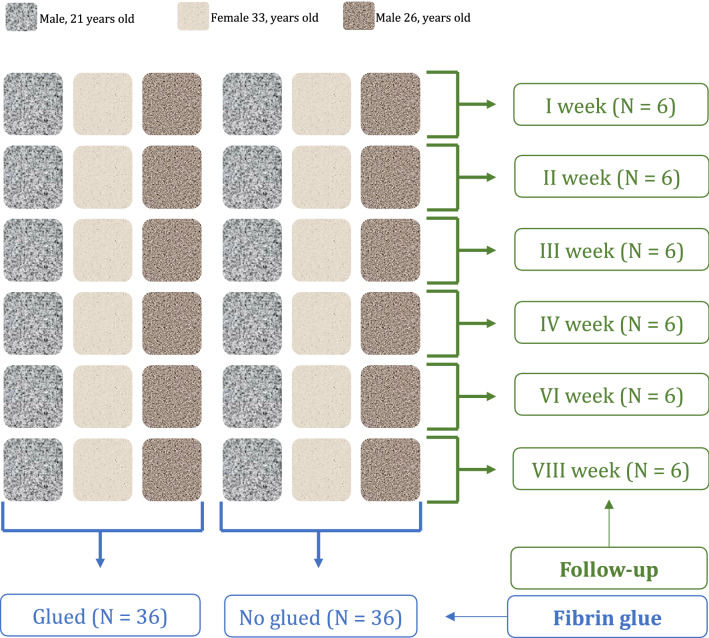
Experimental set-up (N = 72).

All procedures were performed in accordance with the relevant guidelines and regulations and approved by the ethical committee of the Medical Faculty of the University RWTH of Aachen (ID EK305-13). Informed consent was obtained from all subjects and/or their legal guardian(s). The methods used in the present study were already published in a previous in vitro study which evaluated the impact of fibrin glue on a chondrocyte loaded collagenic membrane^23^. Briefly, a resorbable collagen I/III porcine derived membrane (Cartimaix-collagen-membrane; Matricel GmbH, Germany) commonly employed in AMIC was used for the experiments. B-MSCs from three different unrelated donors were used: a 21 years old male, a 26 years old male, and a 33 years old female. The membranes were cut into 0.7 cm × 0.7 cm (area 0.49 cm^2^) in a sterile fashion. Overall, 72 membranes were used for the experiments: 36 non-glued and 36 membranes with fibrin glue (Tisseel, Baxter International Inc, Illinois, USA). Cell proliferation and migration was compared at 1-, 2-, 3-, 4-, 6-, and at 8-week follow-up. For each subject at every time point, 10 membrane sections were evaluated. This process is schematised in Fig. 1.

### MSCs processing

BM-MSCs of patients were isolated from the femoral head following the protocol of Li et al.^24^. Briefly, the femoral head was placed in a beaker and fixed with forceps. A cut surface was thoroughly rinsed several times with cell culture medium. The bone marrow in the cut surface was then loosened with forceps and again thoroughly rinsed with cell culture medium. The medium was placed a 50 ml Falcon tube (ThermoFisher Scientific, Waltham, MA, United States) and centrifuged at 1500 rpm for 10 min at room temperature (ca 20 °C). The obtained supernatant was aspirated, the pellet resuspended with cell culture medium, and seeded in a T75 cell culture flask (Thermo Fisher Scientific, Waltham, MA, USA). The next day, the cells were washed thoroughly three times with Phosphate-buffered saline (PBS) to remove the erythrocytes and other unwanted components. In that session, the medium suspension was also changed. To verify cell properties, flow cytometry was performed using the following FITC coupled anti human antibodies (all from Biorad, USA). Antibody anti CD19 (MCA2495F**)**, CD34 (MCA547F), CD45 (MCA87F) were used as negative control, and CD 73 (MCA6068F), CD90 (MCA90F**)** and CD105 (MCA1557F) as positive control. The obtained data were processed using the software Cyflogic (CyFlo Ltd, Turku, Finland). The membranes were glued to the bottom of a 48-well cell culture dish by pipetting the glue (approx. 10 µL) to the bottom of the well, and the membrane was placed on top. Each membrane was seeded with B-MSCs on the porous side. Following trypsinization (Sigma-Aldrich/Merck KGaA, Darmstadt, Germany) and centrifugation (1500 rmp, 10 min), B-MSCs (third passage) were resuspended in a volume of 40 µl per membrane, and spread as homogenously as possible over the membranes at a density per membrane of approximately 100,000 B-MSCs per cm^2^. After cultivation for 2 h at 37 °C, 5% CO_2_ and a humidity of 90% in the incubator, the wells were filled up with cell culture medium. The cell culture medium was composed as follows: Dulbecco's Modified Eagle's Medium (DMEM) combined with 1 g/l d-Glucose (GlutaMax, low glucose, Gibco/Life Technologies, Paisley, UK), 10% fetal calf serum (FCS, Pan-Biotech, Aidenbach, Germany), 1% penicillin–streptomycin (Pen/Strep, Sigma-Aldrich/Merck KGaA, Darmstadt, Germany), and 1% Minimum Essential Media (MEM) combined with Non-Essential Amino Acids (Gibco/Life Technologies, Grand Island, NY, USA). The medium was changed every 3 days.

### Experiments

At 1-, 2-, 3-, 4-, 6-, and at 8-weeks after seeding, a membrane was fixed in 4% paraformaldehyde (Merck Schuchardt OHG, Hohenbrunn, Germany) for 12 h. Afterwards, the membranes were dehydrated in an ascending alcohol series (1 h per cuvette) as follow: 70% ethanol, 80% ethanol, 96% ethanol, 100% ethanol (2×), and xylene (3×). Subsequently, the membranes were embedded in paraffin (Sakura Finetek Europe B.V., Alphen aan den Rijn, Netherlands) and cooled to − 10 °C. 3 µm sized cuts were prepared on a microtome (Schlittenmikrotom PFM Slide 4003E, PFM Medical AG, Cologne, Germany). To allow better adherence on the specimen slides, the cuts were heated at 60 °C for an hour. The paraffin of the slices was removed with xylol (Otto Fischar GmbH&Co KG, Saarbrücken, Germany) and afterwards the slices were carefully rehydrated with a descending alcohol series as follow: xylene (3×), 100% ethanol (2×), 96% ethanol, 80% ethanol, 70% ethanol, distilled water (5 min per cuvette). The membranes were embedded in Mounting Medium with DAPI (ABCAM, Cambridge, UK) and photographed on the fluorescence microscope (DM/RX, Leica, Wetzlar, Germany). The DAPI contained in the mounting medium ties the DNA of the cell nucleus and emits a blue fluorescence, allowing to detect how the cells in the membrane have spread.

### Outcomes of interest

The outcomes of interest were (1) to evaluate cell migration and (2) cell proliferation within the porous membrane layer. DAPI/nuclei signals were analysed with fluorescence microscope at 100-fold magnification and the software Image J version 1.51 (National Institutes of Health, US). Migration was expressed as the percent of cell ingrowth within the overall thickness of the membrane. The cells which migrated in the deepest layer of the membrane was used a reference. Proliferation refers to the number of cells per mm^3^.

### Statistics

The statistics analyses were performed using the IBM SPSS Statistics version 28.0 (IBM Corporation, Armonk NY, USA). The Shapiro–Wilk test was performed to investigate data distribution. For normally distributed variables the t-test (Welch) was used, the Mann Whitney U test for non-parametric data.

### Ethical approval

The present study was approved by the ethical committee of the Medical Faculty of the University RWTH of Aachen (ID EK305-13).

## Results

### Flow cytometry of isolated B-MSCs

**Figure 2:**
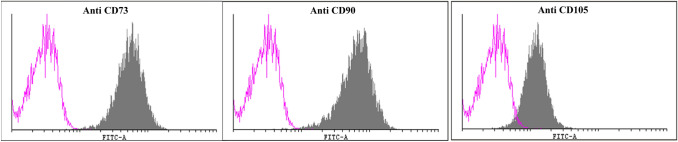
Flowcytometric evaluation of isolated B-MSCs with positive controls of CD73, CD90, and CD105.

The isolated B-MSCs showed a clear positive FITC signal in flowcytometry histogram overlays using the Cyflogic software (Fig. 2). Negative controls of CD 19, CD34, and CD45 showed no positive FITC signal.

### Migration

**Figure 3:**
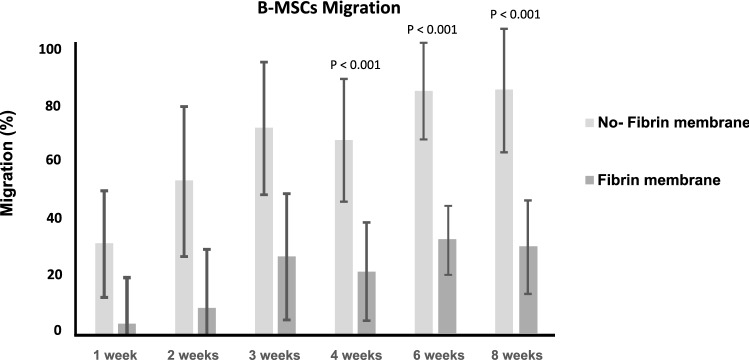
B-MSCs migration within the membrane.

The group without fibrin glue demonstrated greater migration of the B-MSCs within the membrane at week 4 (P < 0.001), 6 (P < 0.001), and 8 (P < 0.001). No difference was found at week 1 (P = 0.4), 2 (P = 0.09), and 3 (P = 0.06). Figure 3 shows the results of cell migration at each follow-up.

### Proliferation

**Figure 4:**
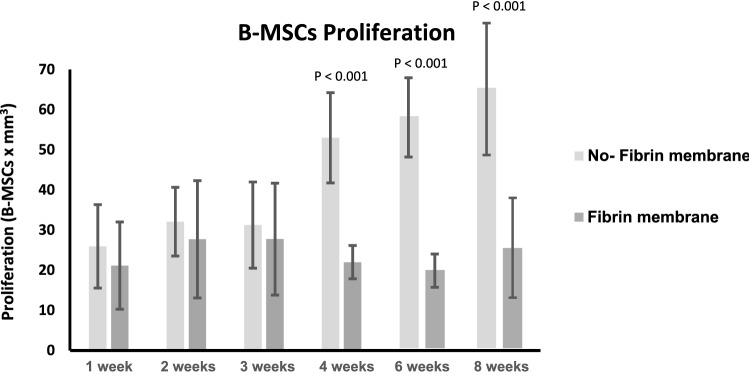
B-MSCs proliferation within the membrane.

The group without fibrin glue demonstrated greater proliferation of B-MSCs within the membrane. These differences were significant at week 4 (P < 0.0001), 6 (P < 0.0001), 8 (P < 0.0001). No difference was found at week 1 (P = 0.7), 2 (P = 0.8), 3 (P = 0.7). Figure 4 shows the results of cell proliferation at each follow-up.

## Discussion

**Figure 5:**
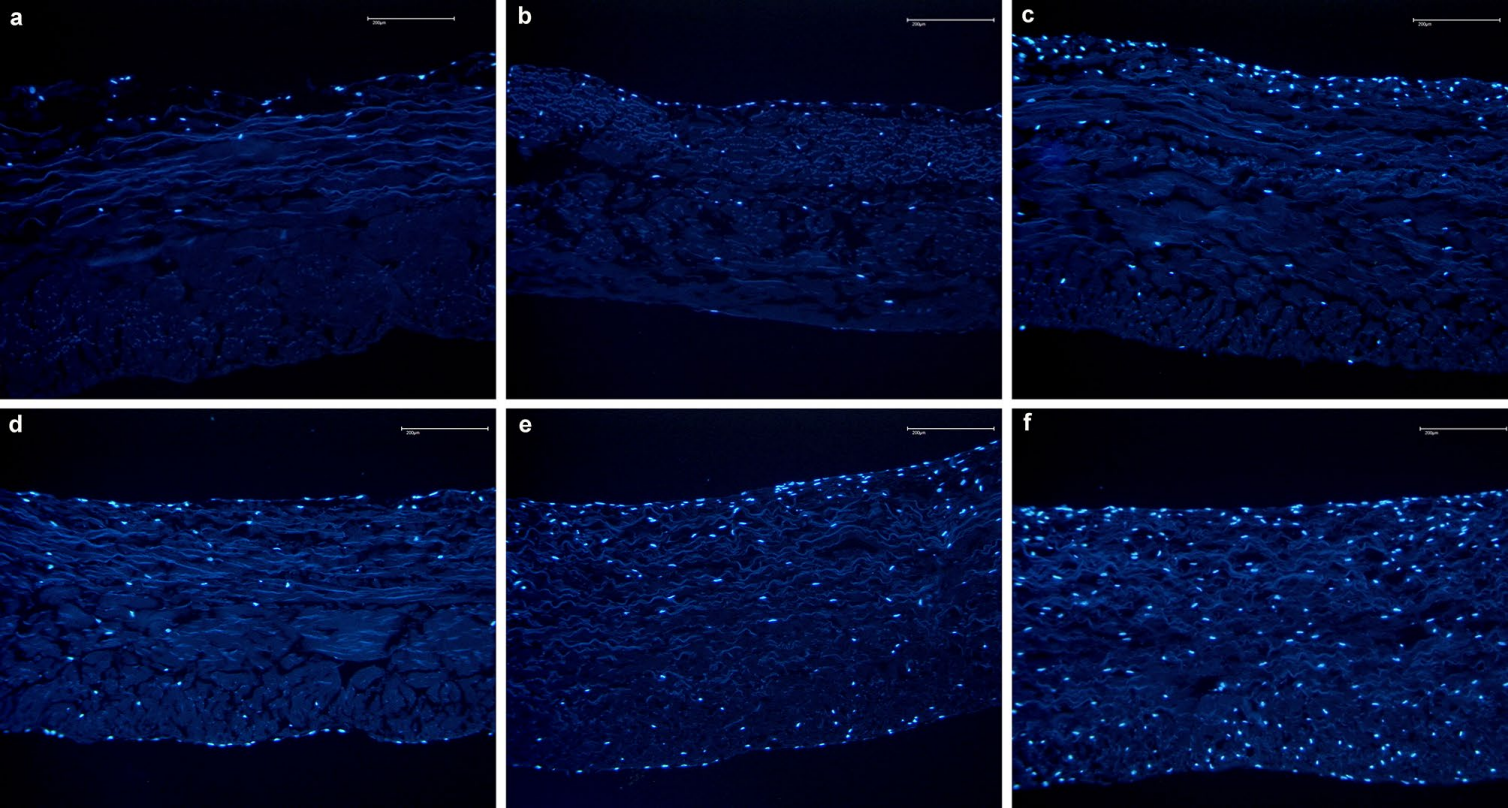
Proliferation and migration of B-MSCs in the membrane without fibrin using the DAPI stained cell nuclei of B-MSCs (blue) assay. Time dependent proliferation and migration of B-MSCs into the non-fibrin treated membrane over 1, 2, 3, 4, 6, and 8 weeks (pictures **a**–**f**, respectively).

**Figure 6:**
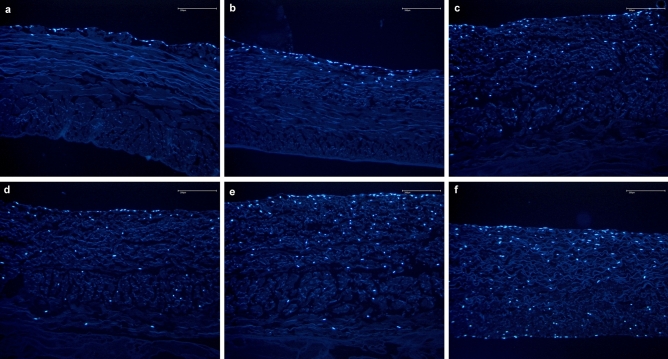
Proliferation and migration in the fibrin glued membrane using the DAPI stained cell nuclei of B-MSCs (blue) assay. Time dependent proliferation and migration of B-MSCs into the non-fibrin treated membrane over the 1, 2, 3, 4, 6, and 8 weeks (pictures **a**–**f**, respectively).

According to the main findings of the present in vitro study, the use of fibrin glue impairs B-MSCs proliferation and migration in a resorbable collagenic biomembrane routinely used in clinical practice (Figs. 5, 6). The no-glue membrane demonstrated in vitro greater migration of the B-MSCs within the membrane at week 4, 6, and 8 weeks and greater proliferation at all these follow-up times.

Given their limited survival and unfavourable intra-articular environment which impairs cell colonisation, isolated microfractures of the subchondral bone are insufficient for cell engraftment^25^. Therefore, the membrane is supposed to protect the blood clot. The ideal properties of such membranes are biocompatibility and biodegradability, sufficient porosity to allow cell penetration, and permeability to permit both gas and nutrients delivery^25^. In addition, an ideal scaffold should permit extracellular matrix formation and the transmission of signalling molecules^26^. In the present experiment, we used resorbable collagen I/III porcine derived membranes which are commonly employed in chondral procedures^22^.

Collagen is the most abundant protein in the animal body^27^. It is a potential biomaterial scaffold for different tissue engineering applications^27^. Collagen is mechanically stable and has a high tensile strength. Different mechanisms are involved in the attachment of cells to collagens: Integrins play a key role in cell attachment^27^. The ∝ 1ß1 and ∝ 2ß1 integrins are the most important collagen binding integrins. ∝ 2ß1 has a high affinity for the fibrillar type I collagen, which is the major constituent protein of bone^28^. The ∝ 2ß1 integrin interaction with type I collagen signals the induction of both the osteoblastic differentiation and matrix mineralization^28^. Also, it was observed that ∝ 2ß1 integrin specific collagen-mimetic surfaces supports osteoblastic differentiation^29^. Altogether, a collagen scaffold offers the natural extracellular matrix environment with an intricate biochemical interplay, which promotes the proliferation and migration of B-MSCs^27^.

Several surgical procedures aiming to repair/regenerate chondral defects include the use of a biological resorbable membrane. Typically, during these procedures the membrane is fixed using sutures. This has been demonstrated to cause irreversible damage to the cartilage, which may lead to chronic pain and premature osteoarthritis^30,31^. Therefore, fibrin glue has been introduced to secure the membrane into the chondral defect. Fibrin is a tissue-derived natural component involved in the clotting process of blood^25^. Fibrin glue was initially employed for haemostasis on wounds by Bergel^32^. Since then, given its biological sealing, haemostatic, and adhesive proprieties, it has been widely used in various surgical fields^33–36^. Commercially available fibrin glues are prepared from allogeneic pooled plasma^37^. Fibrin glue commonly consists of a highly concentrated fibrinogen complex including fibrinogen, fibronectin, factor XIII, and plasminogen, and a high-potency thrombin^34,38^. Fibrin glues have been used for cell delivery, especially keratinocytes and fibroblasts^39,40^. Mainly through the action of thrombin, fibrin glue is believed to promote a variety of cellular responses, increasing cell migration, proliferation and survival^41–45^. However, in the present in vitro study, the addition of fibrin to a commercially resorbable biomembrane impaired B-MSCs proliferation and migration.

The exact mechanism of fibrin glue and its effect on MSC in vitro are largely unknown. In physiological tissue repair following damage, the platelet system is activated, and a fibrin matrix is formed after vasoconstriction and platelet clotting. Inflammatory cytokines, such as interleukin-1ß, tumour necrosis factor-α, and interferon-γ, are released by activated platelets^46^. However, in the present in vitro study, the addition of fibrin in a commercially resorbable biomembrane impaired B-MSCs proliferation and migration. Previous studies investigated the role of fibrin glue on colonic anastomoses in rats and demonstrated compromised healing following fibrin glue sealing^47^. Fibrin is presumed to impede macrophage migration^48^ and phagocytosis of bacteria by neutrophils^49^. We hypothesize that an increased inflammatory activity and immune response is promoted by fibrin glue, in accordance with earlier findings suggesting that fibrin can undergo degradative or inflammatory responses^50^. Previously, reduced collagen concentration caused by an increased production of collagenase from the inflammatory activity induced by fibrin glue has been reported^51^. Moreover, fibrin glue is supposed to stabilize growth factors, preventing the natural enzymatic degrading process^52^. Yet, a negative effect of high concentrations of growth factors in the production of neocartilage in vitro has been demonstrated^53^. So far, the in vitro concentrations of growth factors and their effects on prolifeating tissues have not been further examined^53^. Fibrin glue contains a high concentration of clotting factors but only a low number of stimulating factors^54^. Also, fibrin sealants might impair the migration of chondrocytes via a barrier effect^55^. Thus, it is possible to assumed that the proteins, cytokines, and B-MSC progenitor cells, which can be found in the joint intra-articular environment, might be enabled to proceed to the chondral surface. However, to date, their effect on cartilage repair in vitro and in vivo is not completely understood^56^.

A major disadvantage of fibrin glue is its difficulty in maintaining structural integrity, increasing instability and solubility over time from fibrinolysis^57^. Therefore, by 8 weeks, fibrin gel shrinkage and low mechanical stiffness might have contributed to the fact that fibrin in a resorbable collagenic biomembrane impaired B-MSCs proliferation and migration in our study.

This study certainly has limitations. The limited number of B-œ∆MSCs donors may negatively impact the reliability of the conclusion and increases the risk of publication bias. Moreover, the follow-up is limited to 8 weeks, thus impairing the capability to identify long term cells proliferation and migration. In such unperturbed experimental setting, cells migration and proliferation may not be realistic but preferred regions of migration creating higher density and therefore a false higher proliferation rate could be excluded to a degree by the highly standardized setting of the experiments. By definition, the intra-articular joint environment is complex, with many cell types, proteins, cytokines, solutions, and is subjected to heterogeneous pressures, contacts, and tensions. These factors may influence B-MSC migration, proliferation, and activity. Whether this membrane should be additionally fixed with fibrin, and how it interacts with B-MSCs proliferation and migration still remains unclear. Whether fibrin could promote macro- or microscopical structural changes in the collagenic membrane was not investigated in the present study. However, there were no evident alterations in the structure of the membranes at 8 weeks, suggesting no cytotoxic effect of the fibrin glue. The BMSCs used for the experiments were harvested from patients who underwent total hip arthroplasty for idiopathic joint osteoarthritis and agreed to donate their femoral head for this study. This procedure has been also validated in previous studies^58–60^. However, whether osteoarthritis affects B-MSCs migration and proliferation in our experimental setting is unknown. In addition, BMSCs harvested from the femoral head may be less active than those derived from the iliac crest or vertebrae in growth kinetics and chondrogenic differentiation^61^. We are aware that, given some evident limitations, such experimental study could not satisfactorily clarify these controversies; however, this work may contribute to develop future studies, and overcome current limitation to clinical translation.

## Conclusion

In vitro, the use of fibrin in a resorbable collagen biomembrane impairs B-MSCs proliferation and migration.

## Data Availability

The datasets generated during and/or analysed during the current study are available throughout the manuscript.
